# Zhuang-Gu-Fang Treats Osteoporosis in Ovariectomized Rats by Increasing the Osteogenesis-Related Factors Leptin, Ghrelin, and PYY

**DOI:** 10.1155/2020/8164064

**Published:** 2020-11-17

**Authors:** Yuanjin Chen, Rui Bai, Wenhui Chen, Shuanglei Li, Yunxia Jiang

**Affiliations:** ^1^Graduate School, Guangxi University of Chinese Medicine, Nanning, Guangxi 530001, China; ^2^Department of Medical, Faculty of Chinese Medicine Science Guangxi University of Chinese Medicine, Nanning, Guangxi 530222, China; ^3^Department of Endocrine, The First Affiliated Hospital of Guangxi University of Chinese Medicine, Nanning, Guangxi 530023, China

## Abstract

Zhuang-Gu-Fang is a Chinese medicinal compound mixture, which is mainly composed of traditional remedies like the Epimedium Herb, *Astragalus*, and *Eucommia* among many others. The study is aimed at investigating the therapeutic effect of Zhuang-Gu-Fang in ovariectomized rats. Fifty six-month-old Wistar rats were randomly selected and divided into 5 groups (*n* = 10), namely, model group, positive group, low-dose Chinese medicine group, medium-dose group, and high-dose group. Another 10 sham operation Wistar rats were taken as a negative control group. After 3 months of intervention, the bone mineral density (BMD), procollagen type I N-peptide (PINP), beta C-terminal cross-linked telopeptides of type I collagen carboxyl-terminal peptide (*β*-CTX), Leptin, Ghrelin, and Peptide YY (PYY) of each group were measured. Besides, the ultrastructure of bone structure and osteoblasts was also observed by transmission electron microscopy. Western blot method was used to detect the expression levels of Leptin and Ghrelin in bone tissue, and RT-PCR detected the mRNA expression levels of Leptin and Ghrelin. BMD test indicated that Zhuang-Gu-Fang could effectively prevent the loss of tibia bone in ovariectomized rats. Histomorphology analysis showed that Zhuang-Gu-Fang could preserve trabecular bone structure integrity and improve osteoblast ultrastructure. Notably, the study found out that Zhuang-Gu-Fang worked through balancing the bone metabolism via increasing bone formation/resorption ratio. Additionally, Zhuang-Gu-Fang highlighted the recovery effects in multiple levels of osteogenesis- and osteanagenesis-related factors Leptin, Ghrelin, and PYY. Conclusively, the study proved the therapeutic potential of the Zhuang-Gu-Fang for postmenopausal osteoporosis (PMOP) and further revealed that its therapeutic effect was related to the balance of bone metabolism and the recovery effects of bone-related factors Leptin, Ghrelin, and PYY.

## 1. Introduction

The Statement of the Consensus Development Conference of the National Institutes of Health mentioned that osteoporosis (OP) was a severe global skeletal disorder. The characteristics of the disorder are diminished bone strength, low BMD, loss of bone function, and high risk of fracture [1]. Apart from these classical symptoms caused by the disorder, the senescence-associated OP also couples with several devastating comorbidities, such as age-related neurodegenerative disease, diabetes, and cardio- and cerebrovascular diseases [2]. Thus, they create more physical, psychological, and healthcare burdens and impair diagnosis efficiency.

Menopause is a natural part of aging, and it encompasses the loss of ovarian reproductive function among women. In postmenopausal women, bone mineral density decreases rapidly due to estrogen depletion, which leads to higher fracture risk and higher morbidity compared to men of the same age [3]. The National Osteoporosis Foundation (NOF) estimates [4] that more than 70% of the osteoporosis leading fracture occurred among the patients aged fifty years and above, the majority being postmenopausal women. As a consequence, it is urgent to find a comprehensive and effective approach to prevent postmenopausal osteoporosis.

The study concluded that Leptin, Ghrelin, and PYY had essential roles in gastrointestinal energy metabolism and were also related to bone metabolism closely. Leptin is a protein hormone secreted by fat cells, mainly produced by white adipose tissue. Leptin has a wide range of biological effects, of which the most important is the metabolic regulation center of the hypothalamus. This plays a role in suppressing appetite, reducing energy intake, increasing energy consumption, and inhibiting fat synthesis. Previous studies had shown that Leptin could indirectly inhibit bone formation centrally [5–7]. However, Leptin treatment for mice with a bone loss after ovariectomy (OVX) could reduce the trabecular bone loss, trabecular structure changes, and periosteal bone formation more effective than estrogen, thus, indicating that Leptin could participate in bone reconstruction [8]. In vitro experiments proved that Leptin could directly promote the differentiation and maturation of osteoblast cell lines [9]. Furthermore, Leptin might promote the differentiation of osteoblasts by inhibiting GSK-3*β* [10].

Ghrelin is a peptide hormone composed of 28 amino acids. It is an endogenous ligand of the growth hormone secretagogue receptor. It plays the role of stimulating the release of pituitary growth hormone, stimulating food intake, and regulating energy metabolism and other neuroendocrine, cardiovascular, reproductive, and immune systems [11, 12]. Ghrelin also plays a vital role in bone metabolism. Ghrelin promotes the proliferation and differentiation of osteoblasts through GHSR-1a and can increase the bone density of rats and promote bone formation [13–15].

Further research concluded that Ghrelin increased bone mass through the central mechanism of appetite regulation and might inhibit osteoblast apoptosis through the ERK-AKT pathway [16]. Additionally, a neuropeptide, peptide YY, had been found to play a significant role in bone metabolism in premenopausal women in recent years [17] and could also be used as a link between viscera and bone biology to regulate bone remodeling in rats [18]. Newfoundland population survey indicated that PYY and Ghrelin were bone protective factors [19]. Therefore, we proposed the hypothesis that Zhuang-Gu-Fang may interfere with the process of bone metabolism by regulating Leptin, Ghrelin, and PYY to prevent osteoporosis further.

Zhuang-Gu-Fang is the experience therapy by Professor Li Shuanglei, a famous Chinese medicine doctor in Guangxi. In long-term clinical application, it had been proven that Zhuang-Gu-Fang could effectively improve the clinical symptoms of osteoporosis patients and promotes bone mass increase to a certain extent. Previous animal experiments also confirmed the early intervention effect of Zhuang-Gu-Fang on bone metabolism disorders [20]. This study aims to review further the effect of Zhuang-Gu-Fang on the ultrastructure of osteoblasts, bone metabolism balance, and serum Leptin, Ghrelin, and PYY concentration in ovariectomized rats and explore the treatment of postmenopausal osteoporosis with Zhuang-Gu-Fang-related mechanisms.

Considering the limitations in current PMOP treatment, it is essential to perform a systematic rat model for evaluating the therapeutic effect of the Chinese medicine compound mixture, Zhuang-Gu-Fang. The study used 6-month-old Wistar rats as the model. Then, these traditional Chinese medicines were purified and combined, including Epimedium Herb, Radix Astragali, *Eucommia*, *Dioscorea opposita*, *Salvia miltiorrhiza,* and *Panax notoginseng* to obtain the formula. Furthermore, the study assessed the BMD, bone ultrastructure, and bone histomorphology change after the Zhuang-Gu-Fang compound treatment. More importantly, bone formation and bone resorption capability were detected by RT-PCR, Western blot, and serum analysis to elucidate the therapeutic potential.

## 2. Materials and Methods

### 2.1. Zhuang-Gu-Fang Aqueous Preparation

Zhuang-Gu-Fang consists of six traditional Chinese herbs as shown in Table 1. All herbs were purchased from the First Affiliated Hospital of Guangxi University of Chinese Medicine (Nanning, China) and carefully authenticated by Dr. Hui Tian of Guangxi University of Chinese Medicine (Nanning, China). Voucher specimens (numbers are listed in Table 1) were deposited at the Herbarium of Guangxi University of Chinese Medicine (SDU.TCM, Nanning, China). After drying, all herbs were mixed in proportion; at 105 g, this mixed material was combined with 200 ml of distilled water and boiled for 1 hour at 100^o^. Filtering the decoction, the residual herbs were boiled in water following the same procedure once more. Finally, the two decoctions were mixed and concentrated at 50 ml. Estradiol valerate was provided by the Guangzhou branch of Bayer Medical & Health Co., Ltd. Batch no. 180A.

### 2.2. Animals Model

Sixty 6-month-old female Wistar rats were purchased from SPF Laboratory Animal Technology Co. Ltd. They were housed in an environmentally controlled room, which had a constant temperature (22 ± 1°C) and humidity (55 ± 5%) on a 12-hour light/dark cycle according to the national standard GB 14925–2010 (China). All rats were fed on the standard diet and water regularly. All experiments were carried out per the National Institutes of Health Guide for the Care and Use of Laboratory Animals (NIH Publications No. 8023, revised 1978). The experimental protocols were subject to approval by the Animal Ethics Committee of the Guangxi University of Chinese Medicine.

The design of animal experiments should follow the principle of “3R”, including the replacement, reduction, and refinement of experimental animals. Therefore, the number 10 per group was in line with the statistical requirements. First, the study started with taking the 50 Wistar rats for ovariectomy. The rats were anesthetized with 2% sodium pentobarbital. Then, a longitudinal incision about 2 cm in length was made on the second lumbar vertebra of the midline of the abdomen and back. The incision was separated into the abdominal cavity layer by layer. The ovaries were located along the fallopian tube and pulled out with flat forceps. Bilateral ovaries were removed completely. Another 10 Wistar rats were taken for the sham operation. In the sham operation, only the same amount of adipose tissue around the ovaries was removed. However, the ovaries were not removed, and the fallopian tubes were not ligated. Also, the abdominal cavity was closed with the sutures layer by layer. The rats recovered for one week after the operation, and we took blood samples to determine the estrogen level of all experimental rats before proceeding to subsequent experiments.

The results showed that the estrogen level of ovariectomized rats was significantly decreased, but the estrogen level of the sham operation group was not significantly decreased. Thus, this verified the success of OVX in rats. Fifty successfully modeled rats were divided into 5 groups, 10 rats for each group, which were given equivalent normal saline as the model control group (MC), Zhuang-Gu-Fang 0.5 g/kg per day as the low-dose group (LD), Zhuang-Gu-Fang 1 g/kg per day as the medium-dose group (MD), Zhuang-Gu-Fang 2 g/kg per day as the high-dose group (HD), and estradiol valerate 10 *μ*g/kg per day as the positive control group (PC). Another 10 sham operation rats of the same age were taken as the negative control group (NC). All groups were treated for 3 months.

### 2.3. Bone Mineral Density (BMD) Test

The BMD of each rat was tested once by dual-energy X-ray absorptiometry (DXA) (Hologic Int., USA) before and after 3 months of treatment.

### 2.4. Bone Tissue Collection and Osteoblast Ultrastructure Test

Rats were lightly anesthetized with ether (inhalation, 30 s) and sacrificed by cervical dislocation after 3 months of treatment. One-third of the tibia was taken from the proximal metaphysis of the tibia. The attached muscles and connective tissue were removed and sawed along the median coronal plane. The bone marrow cavity was exposed and sawed into small pieces and fixed in a 4°C fixative solution for 22 hours. Then, it was washed with 0.1 mol/L phosphate-buffered saline (PBS) and decalcified in bone demineralization liquid at room temperature. After decalcifying for 8 weeks, the tissues were trimmed into 1 mm × 1 mm × 1 mm, washed with 0.1 mol/L PBS, and fixed in 3% glutaraldehyde for 2 hours. Subsequently, they are fixed with 1% osmic acid for 2 hours, then dehydrated in 75%, 80%, 95%, and 100% alcohol, embedded in paraffin, and made into the pathological section. After double staining with uranium acetate and lead citrate, the ultrastructure of bone tissues was observed under electronic transmission microscopy (Hitachi-H7650, Japan).

### 2.5. Western Blot Assay

Bone tissue proteins were extracted through RIPA buffer (Thermo Scientific, USA) with a 1% protease inhibitor cocktail (MCE, USA). Protein sample concentrations were measured by Coomassie brilliant blue protein kit. All the Western blot analyses were performed with standard protocol. The blots were detected with primary antibodies as follows: Tubulin (1: 2000; Santa Cruz, USA), Leptin (1: 500; Abcam, USA), and Ghrelin (1: 500; Santa Cruz, USA) and then detected by secondary antibody HRP-anti-mouse or HRP-anti-rabbit (1: 2000; CST, USA). Protein bands were incubated in ECL reagent (Solarbio, China) for 2 min and then visualized with chemiluminescence instrument (Bio-Rad, USA). Protein levels were analyzed by ImageJ software.

### 2.6. Serum Analysis

After 3 months of treatment, blood was collected through the ventral aorta. The serum was obtained by centrifugation. Leptin, Ghrelin, PYY, procollagen type I N-peptide (PINP), and beta C-terminal cross-linked telopeptides of type I collagen carboxyl-terminal peptide (*β*-CTX) level in serum were measured by standard ELISA kit (R&D Int., USA), respectively, following instructions.

### 2.7. RNA Isolation and RT-PCR Assay

The bone tissues which weigh 100 mg–200 mg were ground into a powder with liquid nitrogen. Total RNA was isolated from bone tissue using an RNA isolation kit (Transgene, China) following the manufacturer's instructions. The total RNA which weigh 2 *μ*g was reverse-transcribed by a cDNA transcription kit (Thermo Fisher Scientific, USA). RT-PCR was executed by using SYBR Green I Master on LightCycler®480 (ROCHE, USA) instrument with primers (RT-PCR detection primer sequence was shown in Table 2). 18S rRNA was used as a normalized control. The mRNA levels of target genes were calculated with the ΔΔct method compared to normalized control.

### 2.8. Statistical Analysis

All values were performed by SPSS 16.0 (SPSS Inc., USA) statistical package. All data were in accordance with normal distribution by the Shapiro-Wilk test, and the numerical data are shown as mean ± SEM. The normal distribution data among multiple groups were compared by one-way analysis of variance (ANOVA), and the SNK-q test was used for pairwise comparison. *p* < 0.05 was considered statistically significant.

## 3. Results

### 3.1. Bone Mineral Density (BMD) Increased Capacity of Zhuang-Gu-Fang (Figure 1)

**Figure 1:**
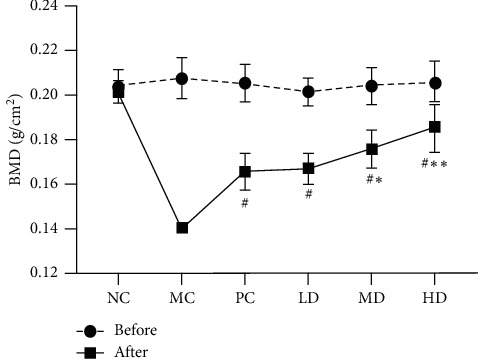
Effect of Zhuang-Gu-Fang on BMD in ovariectomized rats (^*∗*^*p* < 0.05, ^*∗∗*^*p* < 0.01, compared to PC after 3 months of treatment; ^#^*p* < 0.01, compared to MC after 3 months of treatment.).

The BMD of rats in each group was gained before and after 3 months of treatment with Zhuang-Gu-Fang. The BMD data is highlighted in Figure 1. Before treatment, there was no significant difference between each group. However, after 3 months of treatment, the ovariectomized rats were developed, and the BMD of MC group, PC group, LD group, MD group, and HD group all showed a significant decrease compared to the BMD before treatment and NC group (*p* < 0.01). Thus, this demonstrated that bone loss occurred in 3 months. However, after Zhuang-Gu-Fang therapy, the BMD of LD, MD, and HD groups was higher than that of the MC group (*p* < 0.01) in a dose-dependent manner. Moreover, compared to the BMD increased capacity with the PC group (Estradiol valerate 10 *μ*g/kg per day), MD and HD groups exhibited a more significant increase (*p* < 0.05).

### 3.2. Effect of Zhuang-Gu-Fang on Bone Structure and Osteoblast Ultrastructure

**Figure 2:**
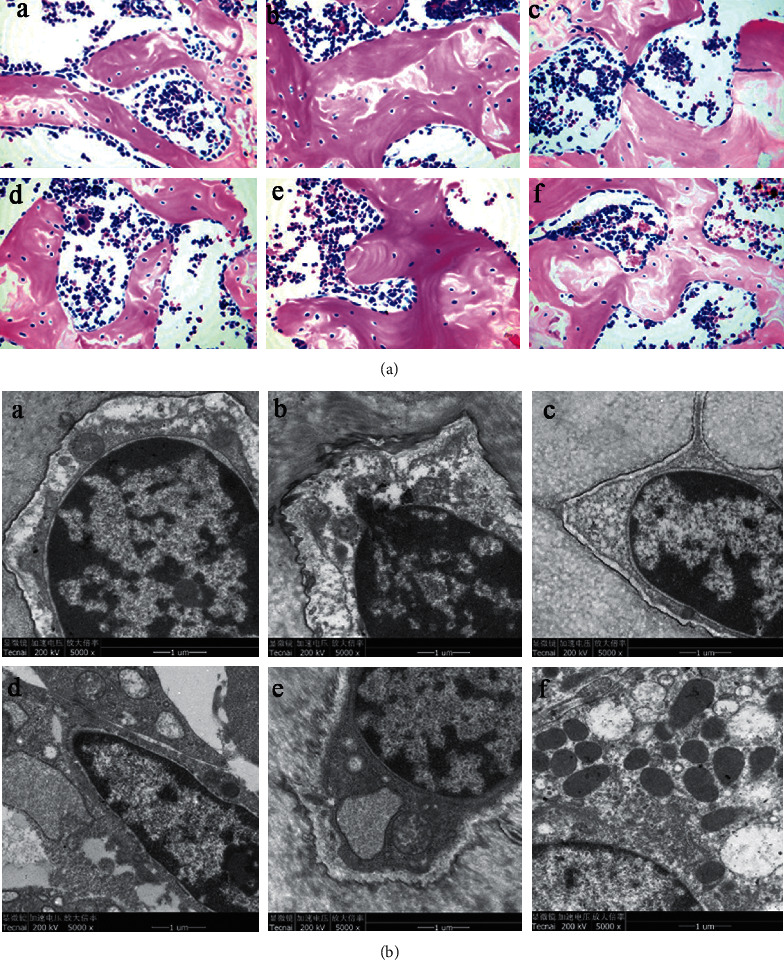
Zhuang-Gu-Fang improves bone structure and osteoblast ultrastructure in ovariectomized rats. (a) HE staining of the tibia bone (×400) of (A) sham operation Wistar rats of the same age which were taken as the negative control (NC) group, (B) model control (MC), (C) estradiol valerate 10 *μ*g/kg per day as the positive control (PC), (D) Zhuang-Gu-Fang low dose (LD), (E) Zhuang-Gu-Fang medium dose (MD), and (F) Zhuang-Gu-Fang high dose (HD) after 3 months of treatment. (b) The osteoblasts ultrastructures were observed by electronic transmission microscopy. These osteoblasts were obtained in (A) NC, (B) MC, (C) PC, (D) LD, (E) MD, and (F) HD groups after 3 months of treatment.

Apart from the BMD increases as a marker of bone-strengthening, the bone structural properties and osteoblast ultrastructure were also considered as a vital factor in bone formation of osteoporosis [21]. To investigate the Zhuang-Gu-Fang effect as above, 1/3 length tibia tissues of each group in rats were collected after 3 months of treatment. Tissue section and HE staining exhibited that the trabecular bone in the sham operation group (NC) was dense, regularly arranged, uniform in thickness, complete in structure, well connected, and small in the bone marrow cavity. Moreover, the osteoblasts proliferated in line with rare osteoclasts (Figure 2(a), A). In the model control group (MC), as the nontherapy ovariectomized rats, the trabecular bone was weak and sparse significantly with a lower amount. Furthermore, it showed uneven thickness, poor connection between parts, and more blind ends of the trabecular and large trabecular gap and bone marrow cavity (Figure 2(a), B). Compared to the model control group (MC), the bone structure of the PC, LD, MD, and HD groups after treatment was more complete, the number of bone trabeculae increased, the arrangement was dense, and the connection was well (Figure 2(a) C–F).

To observe the detailed change of osteoblasts, we adopted electronic transmission microscopy. Zhuang-Gu-Fang could improve the pathological changes in the ultrastructure of osteoblasts. In the model control group (MC), the osteoblast contracted markedly, and its organelles reduced, perinuclear space widened, rough endoplasmic reticulum (RER) expanded, and mitochondria swelled or dissolved (Figure 2(b) B). However, the group of estradiol valerate and Zhuang-Gu-Fang showed a protective effect on the cellular organelles after 3 months of treatment, which appeared as a clear vision of RER and lysosome integrity in PC, LD, MD, and HD groups. In addition, estrogen and traditional Chinese medicine treatment could relieve the expansion of perinuclear space and the swelling of RER and mitochondria (Figure 2(b), C–F).

### 3.3. Effect of Zhuang-Gu-Fang on Balancing Bone Metabolism (Figure 3)

**Figure 3:**
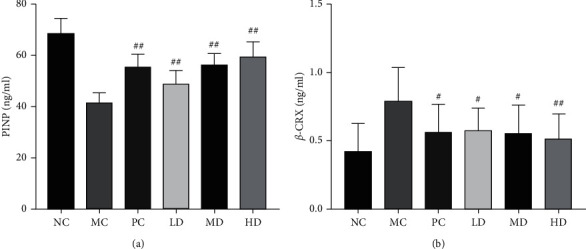
Effect of Zhuang-Gu-Fang on bone metabolic factor in ovariectomized rats after 3 months of treatment (^#^*p* < 0.05, ^##^*p* < 0.01, compared to MC.).

In the previous research, Zhuang-Gu-Fang had shown the protective potential of trabecular architecture and osteoblast ultrastructure against postmenopausal osteoporosis. Moreover, the study observed that Zhuang-Gu-Fang could induce the production of osteoblast and reduce osteoclast. Therefore, the study hypothesized that Zhuang-Gu-Fang might play an essential role in balancing bone metabolism, which regulates the ratio of bone formation and bone resorption process. PINP is the marker of collagen deposition that could reflect the level of bone formation, and *β*-CTX is a specific product of collagen degradation that could reflect the activation of osteoclast and bone resorption [21]. To elucidate Zhuang-Gu-Fang on bone metabolism, we detected PINP and *β*-CTX levels in serum after 3 months of treatment by ELISA kit. As shown in Figure 3, the MC group induced a significant low PINP level and high *β*-CTX level (*p* < 0.01) in serum, whereas LD, MD, and HD groups would increase PINP level and decrease *β*-CTX level. Their effects were similar to those of the PC group. As a result, the bone formation/resorption ratios were increased by Zhuang-Gu-Fang therapy.

### 3.4. Zhuang-Gu-Fang Promotes the Expression of Osteogenesis- and Osteanagenesis-Related Factors Leptin, Ghrelin, and PYY

**Figure 4:**
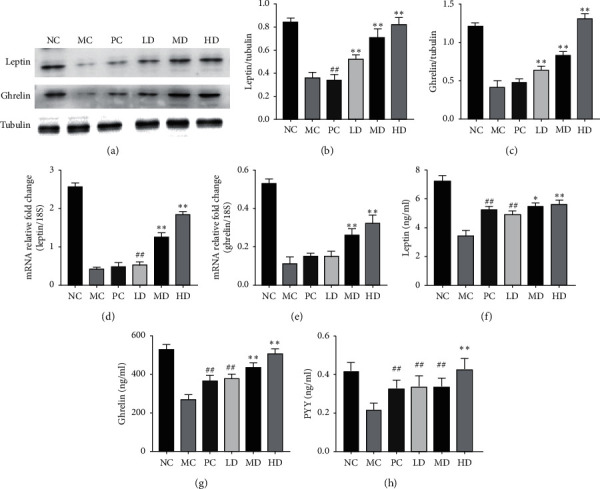
Zhuang-Gu-Fang enhanced the expression of osteogenesis- and osteanagenesis-related factors in ovariectomized rats. (a) Tibia protein samples were prepared and Western blot analyses were performed using Ghrelin, Leptin, and Tubulin antibodies in each rat group after ovariectomized. (b) and (c) Statistical graphs show the relative protein level of Leptin and Ghrelin normalized to Tubulin. (d) and (e) The tibia bone RNA was analyzed by RT-PCR in each group after 3 months of senescence treatment. Bar graphs highlight the mRNA level of Leptin and Ghrelin normalized to 18 S. (f) Leptin, (g) Ghrelin, and (h) PYY levels in serum were also calculated using murine ELISA kit (^*∗*^*p* < 0.05, ^*∗∗*^*p* < 0.01, compared to PC; ^##^*p* < 0.01, compared to MC.).

As to explore whether Zhuang-Gu-Fang could promote osteogenesis, we assessed the protein level of Leptin and Ghrelin via Western blot analysis (Figure 4(a)). The protein level of Leptin and Ghrelin in MC decreased compared with the NC group. The LD, MD, and HD groups exhibited that the Leptin and Ghrelin were markedly higher than those in the PC in a dose-dependent manner (Figures 4(b) and 4(c)). Meanwhile, in relative mRNA expression level, MD and HD groups showed a more significant increase than the PC group (Figures 4(d) and 4(e)). Besides, the study also determined the serum level of Leptin, Ghrelin, and PYY. The concentration of Leptin and Ghrelin in the MC and PC groups markedly decreased compared with the NC group, whereas the MD and HD group could reverse the declining trend (Figures 4(f) and 4(g)). Moreover, the high dose of the Zhuang-Gu-Fang (HD) treatment showed more effectively the capacity of the increasing PYY level in serum than the PC group (Figure 4(h)).

## 4. Discussion

The bone remodeling process included two complex processes of old bone resorption and new bone formation, mainly through two kinds of cells: (1) osteoclasts that promote the resorption of old bones through catabolism and (2) osteoblasts that promote the formation of new bones through anabolic metabolism. Osteoclasts and osteoblasts work together to maintain the balance of osteolysis and bone formation and maintain bone mass at normal levels [22, 23].

Postmenopausal osteoporosis is mainly caused by a sharp drop in estrogen levels after menopause. Studies have shown that with the lack of estrogen in women after menopause, the bone turnover rate is accelerated and the bone metabolism process is negatively balanced. When osteolysis is more remarkable than bone reconstruction, bone loss occurs, the cortical bone becomes thin, and the trabecular bone structure is weak and destroyed, resulting in osteoporosis [24, 25].

As far as it goes, the principal purpose of osteoporosis drugs is enhancing bone mass and strength by reducing bone resorption. In daily fundamental prophylactic treatment, the recommendation of reasonable calcium supplement and Vitamin D intake has been widely accepted in postmenopausal OP remission. The current drugs for the treatment of osteoporosis mainly include RANKL inhibitors and bisphosphonates [26], estrogen or selective estrogen receptor modulators (SERMs) [27], calcitonin [28, 29], strontium salts [30], and parathyroid hormone (PTH) [31]. However, these drugs may cause more adverse reactions, including intestinal related side effects, renal injury, more endocrine disorders and osteonecrosis of the jaw (ONJ), and atypical femoral fractures (ATF) [32]. Our results showed that estrogen supplementation could effectively improve bone loss and increased bone density. However, numerous studies have confirmed that long-term use of hormone replacement or selective estrogen receptor modulators will increase the risk of breast cancer, cardiovascular disease, venous thrombosis, and other diseases, so it is not suitable for long-term clinical application [33, 34].

Notably, compared to the strategies mentioned above, traditional Chinese medicine possesses the unique advantages of protecting bone health in a natural, humane way.

For example, previous studies confirmed that Epimedium and its components could antagonize bone resorption and stimulate bone formation [35] and had a positive effect on the reconstruction of bone biological function [36]. *Astragalus* polysaccharide, the active substance of astragalus, has been proven to effectively inhibit osteoclastogenesis and increase bone mass [37, 38] and prevent osteoporosis in ovariectomized mice [39]. Some studies have found that *Eucommia* can effectively inhibit the reduction of bone density caused by OVX, improve bone strength, effectively improve the obesity caused by estrogen reduction, and have no hyperplastic effect on the uterus [40, 41].

In this study, the research utilized the traditional Chinese medicine Epimedium Herb, Radix Astragali, *Eucommia*, yam, *Salvia miltiorrhiza,* and Tianqi to make up the Zhuang-Gu-Fang. Experiments showed that our formula could largely maintain or increase bone formation so that the balance of bone metabolism was regulated in dual-directions, which is fundamentally different from other current osteoporosis drugs. These views are based on the present results that in ovariectomized rats the Zhuang-Gu-Fang induces (1) a significant BMD increase, (2) bone structure and osteoblast ultrastructure improvement, (3) the balance of the bone metabolism, and (4) enhancing the expression of osteogenesis and osteanagenesis factors Leptin, Ghrelin, and PYY.

Although the cell mechanism of Zhuang-Gu-Fang in the treatment of osteoporosis is not fully understood and appreciated globally, current evidence has indicated the therapeutic potential of this formula in postmenopausal osteoporosis. Therefore, in the next experiment, the study will verify the cell and molecular mechanism of action of this formula and effectively use the advantages of traditional Chinese medicine further to explore the long-term beneficial treatment of postmenopausal osteoporosis.

## 5. Conclusions

In summary, the study can attest that Zhuang-Gu-Fang can improve the BMD of ovariectomized rats, balance bone metabolism, and increase the expression levels of bone metabolism and the recovery effects of bone-related factors Leptin, Ghrelin, and PYY. These results suggested that Zhuang-Gu-Fang could treat postmenopausal osteoporosis, which provided the experimental evidence for PMOP treatment of traditional Chinese medicine.

## Figures and Tables

**Table 1:** Constituents of Zhuang-Gu-Fang.

Components	Amount used (g)	Voucher specimen numbers
*Epimedium sagittatum* (Sieb. et Zucc.) Maxim. Berberidaceae	30	SDU.TCM0047
*Astragalus membranaceus* (Fisch.) Bge. Leguminosae	20	SDU.TCM0025
*Eucommia ulmoides* Oliv. Eucommiaceae	15	SDU.TCM0107
*Dioscorea opposita* Thunb. Dioscoreaceae	15	SDU.TCM0088
*Salvia miltiorrhiza* Bge. Labiatae	15	SDU.TCM0126
*Panax notoginseng* (Burk.) F. H. Chen. Araliaceae	10	SDU.TCM0093

**Table 2:** Information on RT-PCR primers.

Primer name	Primer sequence (5′-- > 3′)	Products
18S-F1	GTCTGTGATGCCCTTAGATG	178 bp
18S-R1	AGCTTATGACCCGCACTTAC	
Rat-Leptin-F1	GTGGAGGGGTAAGAGTCGGA	210 bp
Rat-Leptin-R1	GGTGACCAAGGTGACATAGCG	
Rat-Ghrelin-F2	TCAGCATGCTCTGGATGGAC	109 bp
Rat-Ghrelin-R2	TGCAGTTTAGCTGGTGGCTT	

## Data Availability

All data generated or analyzed during this study are included within the article and available from the corresponding author upon request.
